# Responses of Aquatic Plants to Eutrophication in Rivers: A Revised Conceptual Model

**DOI:** 10.3389/fpls.2018.00451

**Published:** 2018-04-26

**Authors:** Matthew T. O’Hare, Annette Baattrup-Pedersen, Inga Baumgarte, Anna Freeman, Iain D. M. Gunn, Attila N. Lázár, Raeannon Sinclair, Andrew J. Wade, Michael J. Bowes

**Affiliations:** ^1^Freshwater Restoration & Sustainability Group, Water Resources, Centre for Ecology & Hydrology, Edinburgh, United Kingdom; ^2^Section for Stream and Wetland Ecology, Department of Bioscience, Aarhus University, Aarhus, Denmark; ^3^Department of Geography and Environmental Science, University of Reading, Reading, United Kingdom; ^4^Faculty of Engineering and the Environment, University of Southampton, Southampton, United Kingdom; ^5^River Water Quality & Ecology Group, Water Resources, Centre for Ecology & Hydrology, Wallingford, United Kingdom

**Keywords:** nutrient, macrophyte, eutrophication, morphotype, phosphorus

## Abstract

Compared to research on eutrophication in lakes, there has been significantly less work carried out on rivers despite the importance of the topic. However, over the last decade, there has been a surge of interest in the response of aquatic plants to eutrophication in rivers. This is an area of applied research and the work has been driven by the widespread nature of the impacts and the significant opportunities for system remediation. A conceptual model has been put forward to describe how aquatic plants respond to eutrophication. Since the model was created, there have been substantial increases in our understanding of a number of the underlying processes. For example, we now know the threshold nutrient concentrations at which nutrients no longer limit algal growth. We also now know that the physical habitat template of rivers is a primary selector of aquatic plant communities. As such, nutrient enrichment impacts on aquatic plant communities are strongly influenced, both directly and indirectly, by physical habitat. A new conceptual model is proposed that incorporates these findings. The application of the model to management, system remediation, target setting, and our understanding of multi-stressor systems is discussed. We also look to the future and the potential for new numerical models to guide management.

## Introduction

Large riverine aquatic plants or macrophytes, as they are known, are primary producers and can grow submerged below, floating on, or up through the water surface (Pieterse and Murphy, 1990). They are an important constituent of aquatic ecosystems as they directly influence the hydrology and sediment dynamics of river systems through their effects on water flow and play key functions in biogeochemical cycles (French and Chambers, 1996; Chambers et al., 1999). They also provide shelter and refuge (Suren et al., 2000), act as a food source (Gross et al., 2001), and provide a structurally complex environment over spatial scales ranging from millimeters (Dibble et al., 2006) to hundreds of meters (Rennie and Jackson, 2005). Consequently, aquatic macrophytes affect the conservation ecology and the diversity and composition of other biotic assemblages.

As aquatic macrophytes are primary producers, they need light, water, and carbon dioxide to photosynthesize and oxygen to respire (Moss, 1988). They also require micro-nutrients and macro-nutrients, with phosphorus and nitrogen being the key macro-nutrients. Eutrophication is the syndrome associated with an excess of macro-nutrients derived from anthropogenic sources, which leads in turn to excess plant growth and the exclusion of less competitive species.

Eutrophication is not a new problem; it came to prominence in the middle of the last century. In the interim period its impacts have become globally widespread, occurring wherever catchment agriculture is intensive and human populations are dense (Smith, 2003). There is also emerging concern regarding supra-catchment nutrient sources, whereby N is deposited from the atmosphere on otherwise pristine environments with consequent implications for receiving water courses (Vet et al., 2014). As sessile organisms, aquatic macrophytes are subject to a wide range of biotic and abiotic stresses including eutrophication stress derived from upstream catchment processes (Marion et al., 2014). Nutrient enrichment is thought to impact competition for light between aquatic plants and also between the plants and either epiphytic and/or planktonic algae (Hilton et al., 2006). The process is very well described for lakes where nutrients encourage the growth of algae that outcompete littoral macrophytes for light (Moss, 1998). Initially, plants are lost from the deepest water where there is the least light penetration and, as the process worsens, all submerged plants are eventually lost from the system that becomes dominated by algae. This is an undesirable state with the conservation status of the water body in question becoming significantly reduced (Moss, 1998). Once established, the algae-dominated state is stable and it is challenging to flip a system between the two possible alternate stable states that are either macrophyte or algae dominated (Scheffer and van Nes, 2007). In addition to the nutrient levels, the amount of time water is retained (retention time) within a lake is a key factor controlling eutrophication processes.

It has been hypothesized that the process is similar in lakes and rivers and many of the same concepts are transferable; for example, the application of alternate stable states, limiting nutrients, retention time, and light limitation. In rivers, water velocity must also be considered as a significant process control (Hilton et al., 2006). Although significant progress has been made in our understanding of the process of eutrophication in rivers, understanding of plant responses to macro-nutrient enrichment has remained patchy and disparate pockets of research in different fields have yet to coalesce into a consensus view. We discuss how well the lake eutrophication concepts have transferred to rivers and emerging areas of research, and we look forward to the future of this applied field by identifying key areas for research.

## Eutrophication in Rivers – a Decade Ago

There have been a number of reviews of eutrophication focusing on lakes and estuaries and rivers (Carpenter et al., 1998; Smith, 2003; Smith and Schindler, 2009). Some provided a strong focus on primary production in rivers and the importance of understanding threshold responses to nutrient enrichment (Dodds, 2006, 2007) with a comprehensive conceptual model emerging, focusing on eutrophication in rivers (Hilton et al., 2006). This model was applied or used to provide supporting concepts for over 200 papers and can be considered a stimulus for a step change in focused research on the topic. It included a conceptual model which stated that the primary mechanism by which nutrients impacted rivers was by altering competition for light between competitive and non-competitive macrophyte species and epiphytic algae. Competitive macrophytes were considered capable of out-competing other macrophyte species, while they in turn would be out-competed for light by epiphytes. The various additional controls on these interactions were incorporated into a diagram of interactions and included invertebrate grazing on algae and the sloughing effects of water velocity. However, the study was hampered by a lack of empirical data to confirm and quantify the competitive exclusion processes. It was not known, for example, at what nutrient level algal growth in rivers is no longer limiting. Quantitative data are now available and indicate critical differences in the potential form of interactions between algae and submerged macrophytes, leading to a fundamental revision of the original model. We summarize the evidence for the core statements in the original paper and provide a critique below of the key concepts, thereby allowing the model to be updated and revised (see **Table 1** and **Figure 2**).

**Table 1:** A list of predictions and statements on eutrophication processes in rivers from the Hilton et al. (2006) conceptual model and evidence which now supports or contests these statements.

Process	Original Predictions	Evidence
Travel time	Lower river reaches move toward phytoplankton dominance.	Evidence suggests this is the case. Empirical and modeling evidence from the United Kingdom indicates that areas of dead water where phytoplankton numbers can increase act as additional inoculum leading to higher numbers found higher upstream than would otherwise be anticipated from distance downstream (Reynolds, 2000).
Nutrient limitation	Median concentrations of bio-available dissolved nutrients are a more useful predictor of trophic status than load.	This has yet to be tested.
All processes	A large number of interacting parameters make eutrophication complex.	Our knowledge is such that a systematic approach to diagnose eutrophication dynamics is now possible, see **Figures 1, 2**.
Light limitation	The key factor in the loss of macrophytes communities is the development of epiphytic algae which reduce light reaching macrophytes.	The key factor in loss of macrophytes seems more likely to be competition from competitive macrophyte species tolerant of multiple stress with epiphytic algae most important in channels with gentle slopes.
Light limitation	In eutrophic systems, nutrients are rarely limiting but force light limitation.	There is some evidence that macrophytes continue to increase in biomass with increasing nutrient levels but further data is required.
Nutrient limitation	In low-to-medium productive systems nutrients may limit macrophyte growth.	There is evidence that macrophyte biomass is lower in systems with lower P.
Physical habitat and light limitation	Macrophytes which dominate in eutrophic conditions are fast growing or grow well at low light levels.	Submerged macrophytes present at high nutrient levels do have these characteristics, although their trait profiles are more complex; see Baattrup-Pedersen et al. (2015). Also, if hydraulic conditions are suitable, emergent and floating species may persist without functioning well at low light levels.
Physical habitat and light limitation	Rivers subject to high flood flows will show eutrophication effects at lower nutrient levels.	There is no evidence for this.
Physical habitat and light limitation	Qualitative descriptors of different trophic levels in rivers can be based on the succession of plants described by the model. Oligotrophic — strong macrophyte stands with a good representation by submerged plants; Mesotrophic — evidence of slight epiphyte cover and the appearance of benthic algae; Eutrophic—increasingly heavy epiphyte cover with dominance by floating-leaved and emergent plants; Hyper-eutrophic-collapse of macrophyte stands leaving heavy attached filamentous and/or benthic algal cover.	The reality is somewhat different with the physical habitat characteristics determining the potential for different morphotypes in the first instance, see **Figure 1**.
Light limitation	Epiphyte biomass per unit area of macrophyte is a useful monitoring indicator.	This remains untested but has clear potential.
Nutrient limitation	P levels may need to be lowered significantly if we are to see a response in macrophyte communities.	The threshold nutrient values required to see the return of less competitive macrophyte species is not yet known. Equally important is understanding the potential for natural re-colonization from local seedbanks and if additional replanting work is necessary.

There was an assumption that the same competitive dynamics would apply to all macrophyte morphotypes/growth forms. Hence, it was inferred that emergent and floating-leaved species would respond in an analogous manner to nutrient enrichment as submerged species, although there is little opportunity for epiphytic algae to compete with these species. The only opportunity is at the start of the growing season when plants of this type produce fresh shoots from underground storage organs which must then grow up through the water column before the plant produces leaves above the water (Sculthorpe, 1967). At this life stage, these plants are highly resilient to competition and have the capacity to grow to the surface using energy reserves from the overwintering organs. It was hypothesized that emergent and floating-leaved plants would, in all short retention rivers, replace submerged macrophytes. Our understanding of hydraulic habitat requirements now suggests that a more nuanced response, dependent on the physical habitat template, is more probable and that approach is described in detail below (Puijalon et al., 2005, 2011).

In the original conceptual model, the importance of residency time was identified as critical to determining whether a channel would be dominated by benthic or pelagic production. Evidence has emerged that both supports that assertion and suggests that there are important implications for rivers subject to multiple stress, described in detail below. The co-occurrence of eutrophication with other stresses, especially hydromorphological impacts, suggests that future work will need to integrate an understanding of multiple impacts (Johnson and Hering, 2009; Baattrup-Pedersen et al., 2015, 2016). We describe updates to the research agenda to facilitate tackling this challenge. All these developments are synthesized below in a revised conceptual model and the implications for future management are described in detail.

The benefits of targeted data collection have brought increasing clarity to this field of applied research and the point is approaching where effective numerical modeling is possible. We describe the potential application of such models.

## Limiting Nutrients – How Algae Influence Macrophytes

The review by Hilton et al. (2006) was the first to suggest that the rates of periphyton and phytoplankton growth in rivers were the critical drivers of macrophyte community structure and biomass, and other symptoms of eutrophication. Excessive algal growth, both within the water column and particularly on plant leaves, played a key role in controlling the amount of light that was available to the macrophytes, and hence limited energy to drive plant growth. This was seen as the key process that linked excessive nutrient enrichment, loss of submerged macrophytes, and increased dominance of emergent species (as macrophytes that can produce leaves above the water surface are able to harvest sunlight directly, and are largely unaffected by high epiphytic or planktonic algal biomass in the water column).

The critical link to eutrophication was that nutrient enrichment was assumed to increase algal growth rates. Many previous field experiments have supported this assumption. Nutrient diffusing substrata (Francoeur et al., 1999; Tank and Dodds, 2003) and stream enrichment experiments (Greenwood and Rosemond, 2005; Sabater et al., 2005) have shown that increasing nitrate and dissolved phosphorus concentrations (both singly and in combination) can increase attached periphyton growth rates. These approaches have greatly increased ecosystem understanding in low-nutrient environments. However, since the 1990s, nutrient concentrations and P concentrations in particular have decreased markedly in many rivers (Foy, 2007; Bowes et al., 2014) due to improved wastewater treatment processes and tighter control of nutrient leaching from agriculture. These step reductions in P concentrations in particular have not so far delivered the expected reduction in periphyton and phytoplankton biomass and the overturning of algal-dominated to macrophyte-dominated river systems. This is because, despite the reductions in river P concentrations, these nutrients were still in excess and not limiting primary production and algal growth. To determine the P concentrations that needed to be attained in order to reduce algal growth rates, experiments with the capability to reduce P concentrations were developed.

Over recent years, Bowes and co-workers developed within-river flume mesocosms to specifically investigate the relationship between periphyton growth and P concentration, as suggested by Hilton et al. (2006). These flume mesocosms enabled the P concentration of the incoming river water to be both increased (by addition of a concentrated P solution) and decreased (by dosing with an iron solution to precipitate out the dissolved P in the incoming river water). This simultaneously produces a gradient in P concentrations across the flumes, and the impact on periphyton growth rate in each flume can then be determined. This methodology has been applied to nine rivers, covering a wide range of nutrient concentrations, including the relatively pristine river Rede, Northumberland [soluble reactive P (SRP) concentration = 15 μg P L^-1^; McCall et al., 2014], the mesotrophic rivers Kennet and Lambourn (50 μg SRP L^-1^; Bowes et al., 2010; McCall et al., 2017), and the eutrophic river Thames (230 μg SRP L^-1^; Bowes et al., 2012b).

In all of these experiments, significantly increasing SRP concentrations in the river water for sustained periods (usually c. 9 days) did not increase periphyton growth rate or biomass. This is a key finding, and shows that in most nutrient-enriched rivers typically found in the United Kingdom, the process of eutrophication (typified by excessive algal blooms and loss of macrophytes) is not caused by intermittent increases in P. The flume experiments showed that in highly enriched rivers such as the river Thames, SRP concentrations needed to be reduced to below c. 100 μg SRP L^-1^ before biofilms become P limited (Bowes et al., 2012a). Periphyton community structure only shifted toward less nutrient-tolerant species when P concentrations were reduced below 30 μg L^-1^. Similar P thresholds have been observed in national surveys of the trophic state of streams in the United States (Dodds et al., 2002). While there has been a focus on the role of P, N co-limitation has been reported and is considered to be widespread (Dodds, 2006, 2007) and has significant implications for stoichiometric effects on nutrient limitation.

Studies of hourly chlorophyll concentration data for the middle reaches of the river Thames (Untied Kingdom) have shown that chlorophyll concentrations only increase when (1) the water temperature is within a range of 9–19°C, (2) flow is below 30 m^3^ s^-1^, (providing enough residence time to generate significant biomass before being washed into the estuary), (3) there are three or more sunny days of >3 h of sunshine duration, and (4) nutrient concentrations are above limiting levels (Bowes et al., 2016). Phytoplankton blooms commence as a result of the physical conditions (temperature, flow and light) being suitable, and are not caused by increases in nutrient concentration alone. Nutrient concentrations (P and dissolved silicon) potentially play a part in bloom cessation, as they can be reduced to very low, potentially limiting concentrations due to sequestration by the phytoplankton biomass.

## Physical Habitat Template and Light Limitation

The assertion that competition for light is a primary determinant of macrophyte assemblages appears sound. The depth limit for macrophyte growth is when the water transparency allows less than 1–4% of light to reach the plants (Sculthorpe, 1967). Flowering macrophytes are confined to shallower depths than bryophytes and charophytes (Middelboe and Markager, 1997). Rosette growth forms, frequently characterized by a low growth rate, are limited to shallow river reaches (Vestergaard and Sand-Jensen, 2000).

The most dramatic effect of light competition on instream vegetation may not be from phytoplankton or epiphytic algal growth but, in the middle reaches of rivers, can be an effect of dense riparian vegetation shading that can completely exclude macrophytes (Dawson, 1981; Dawson and Hallows, 1983). New work has indicated that in systems where incident light is good, it can require both riparian shading and excessive epiphytic growth before competition for light causes a measurable reduction in macrophytes (Kohler et al., 2010). However, there is conflicting evidence on this topic and it is not clear if riparian and epiphytic shading will always work in concert to have a negative impact on macrophytes. The flume mesocosms used to investigate the impact of nutrient concentration on periphyton growth rates have also been used to investigate the impact of reducing light levels. These experiments showed that reducing light levels by applying shading (to mimic the impact of a full riparian tree canopy) reduced the periphyton growth rate by up to 50% (Bowes et al., 2012b). A similar flume study on the river Lambourn showed that the periphyton were light limited at ambient phosphorus concentrations (49 μg SRP L^-1^), but if the SRP concentration decreased to ≤30 μg L^-1^, the periphyton were co-limited by phosphorus and light. Studies of periphyton biomass at the Hubbard Brook Experimental Forrest, United States (Bernhardt and Likens, 2004) have found similar instances of light limitation, with periphyton accrual rates reducing when trees came into leaf. These observations indicate that increasing riparian tree cover could be an important management tool for controlling excessive periphyton growth, alongside reducing SRP concentrations to potentially limiting concentrations.

To successfully exclude macrophytes by out-competing the higher plants for light, epiphytes must reduce light reaching the plants to a level below that needed for photosynthesis to achieve compensation point (the point at which carbon fixation matches carbon loss through respiration; Spencer and Bowes, 1990). It is notable that *Ranunculus penicillatus*, a dominant rheophilic and competitive species, is quite resistant to light reduction, continuing to grow, albeit very weakly, at c. 20% normal incident light (Dawson and Kern-Hansen, 1978). What is also clear is that this plant is not competitively excluded at high nutrient concentrations, and in fact its biomass continues to increase with nutrient concentration (O’Hare et al., 2010). This is contrary to predictions by Hilton et al. (2006), which indicated that at high nutrient loads, macrophytes will inevitably be excluded.

Light must remain at this reduced level for a period sufficient long that the plant is drained of all stored resources before it will die. If the thickness of the epiphyte layer determines light transmittance and mass transfer to the macrophyte leaf, then factors which interrupt epiphyte growth and inhibit layer development must determine the impact of epiphytes at high nutrient concentrations. In shallow eutrophic lakes evidence suggests grazing invertebrates, and the presence or absence of their predators may play a key role in controlling the switch between alternate stable states (Jones et al., 2002; Jones and Sayer, 2003). In rivers they may play a comparable role, and water shear forces may also be key as the maximal abundance of periphyton is reduced by high flows (Biggs, 1996). In a study on *Ranunculus penicillatus* (O’Hare et al., 2010), field observations indicated that the hydraulic conditions were not suitable for epiphytic algae to completely coat the submerged macrophytes.

It had been suggested that as eutrophication progressed, macrophyte growth forms would be sequentially lost from rivers (Hilton et al., 2006). Initially, submerged macrophytes dominate. As increasing nutrients increase competition for light, submerged species are outcompeted and replaced by growth forms that are better at competing for light (**Figure 2**). This concept, while solid in some respects, insufficiently acknowledged the range of naturally occurring macrophyte assemblages in rivers. The natural condition for all river macrophyte communities is not necessarily dominance by submerged species. Since ancient times there has been an appreciation that the growth form of plants is related to their physical habitat requirements (Sculthorpe, 1967). The ability of a plant to live in a particular hydraulic setting is determined by a number of factors, but crucial aspects are its physical attributes: its shape, its size and its ability to reconfigure (O’Hare, 2015). At its simplest, this means that the broad plant morphotypes recognized by aquatic botanists exhibit different adaptions to hydraulic habitat conditions. In the case of rooted macrophytes, a suitable substrate is also required (Bornette and Puijalon, 2009). Some species require rocky substrates and others are able to anchor themselves firmly in gravels. However, most species require finer substrates. Emergent and submergent species are associated with finer bed sediment, whereas mosses are associated with coarser material. Flume studies have confirmed these broad hydrodynamic relationships in terms of plant form breaking strength, flexibility, and drag, and critical analysis of large datasets on the field distribution of aquatic macrophytes confirm the dominance of different morphotype changes with stream power (Dawson et al., 1999; Gurnell et al., 2010, 2013; O’Hare et al., 2011; Miler et al., 2014). Stream power is a metric used by fluvial geomorphologists and is calculated from the stream slope and discharge representative of common flood conditions. Channel size, sediment character and instream hydraulics all scale-up with stream power. Upland streams, on steep gradients with high stream power, are dominated by bryophytes; moving downstream, stream forces can reduce and submerged macrophytes dominate; then in lowland areas, where stream forces are weakest, emergent and floating-leaved rooted species may dominate.

**FIGURE 1:**
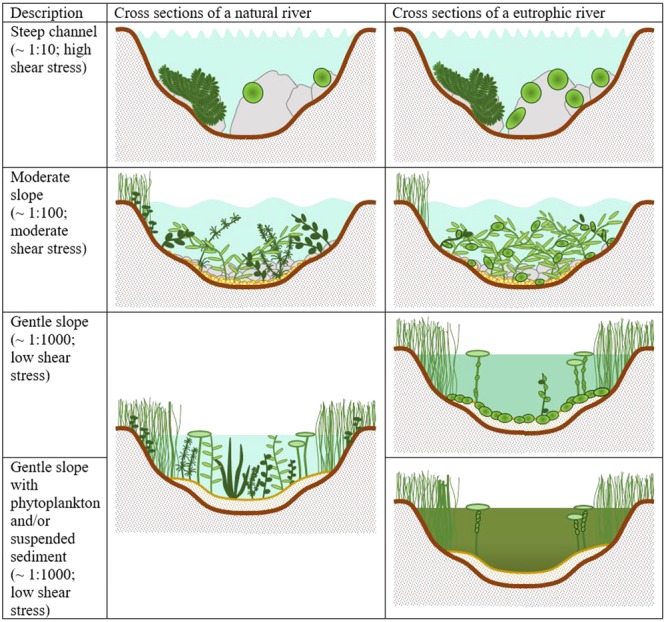
A visual representation of river cross sections on channels of different bed slopes. Typical macrophyte assemblages are indicated under natural and eutrophic conditions.

Hydraulic and hydrological conditions describe the habitat templates against which eutrophication operates. It provides a context and a basis for understanding how, where, and when eutrophication may progress in different ways. We can conclude then that the replacement of one macrophyte morphotype can only proceed where the physical conditions are suitable (**Figure 1**). Those conditions are most likely found in slow flowing margins and low gradient channels where this type of interaction is possible. In other rivers with stronger flows, this kind of replacement is less likely.

**FIGURE 2:**
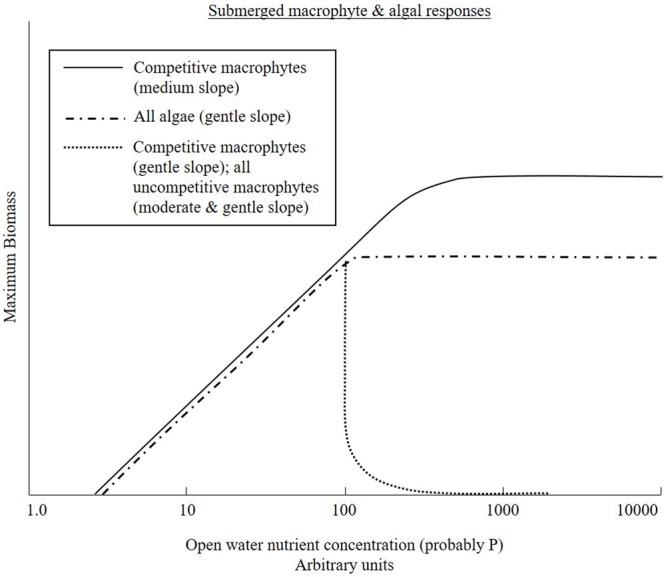
A conceptual diagram adapted from Hilton et al. (2006) indicating the current understanding of primary producer responses to nutrient enrichment.

## Travel Time

Retention time is widely used in lake ecology to understand how eutrophication functions (Moss, 1998). Fundamentally, if the amount of time water resides in a lake is shorter than the doubling time of planktonic algae then the opportunity for nuisance algal blooms to develop is unlikely. In an analogous manner, if it takes water a relatively short time to move down a river from the top of a watershed to the sea, then there is little opportunity for planktonic algae to develop and primary production will either be benthic in nature or allochthonous in origin. Location along the river continuum has a major impact on phytoplankton biomass (Reynolds, 1999, 2000). Headwater streams will always have low phytoplankton concentrations, whereas the lower sections of rivers are often characterized by higher chlorophyll concentrations during the growing season. Classically under the “River Continuum Concept,” rivers widen downstream and therefore are unshaded in the middle of the channel, reducing light limitation and promoting periphyton growth in middle sections (Vannote et al., 1980). Phytoplankton require time to go through multiple reproductive cycles, to build up biomass within the water column and this only manifests as high phytoplankton concentrations in the lower reaches. Therefore, phytoplankton biomass will increase with increasing river length and increasing presence of aggregated dead zones within the river (Reynolds, 2000). Sudden increases in phytoplankton biomass can also occur in streams/rivers downstream of the outlet of lakes (Hilton et al., 2006). The impact of dead zones and other stagnant regions is clearly shown in a study of the river Thames and its tributaries by Bowes et al. (2012a), who showed that the rivers that were connected to canal systems or lock systems had approximately six times higher chlorophyll concentrations than naturally flowing rivers of the same length. Travel time is, therefore, fundamental to how eutrophication will be manifested, either as an excess growth of sessile benthic algae, epiphytic algae, and macrophytes or, alternatively, as excessive planktonic growth. The investigations into the potential of modeling similar transitions has already begun (Hilt et al., 2011).

In summary, there are a number of factors that may influence the point along a channel at which pelagic rather than benthic production is considered dominant. The size of the initial inoculum of phytoplankton is considered a key factor and the addition of impoundments to systems is likely to accelerate the development of phytoplankton. Empirical evidence has also come to light which indicates that channel form has a significant effect on the point at which there is a shift between benthic and pelagic production in river systems. Phytoplankton can build up to higher numbers than expected based on simple measures of travel time. The mechanism at play involves the retention of phytoplankton in areas of slow flowing water, on the outside of meander bends, for example. Here the plankton can build up in numbers and then inoculate the main thalweg flow. This dynamic has a number of interesting implications that are discussed more fully in the section on management of river systems.

## A Revised Conceptual Model

Much of the conceptual model developed by Hilton et al. (2006) is not contested, but significant changes are now possible based on the data and analysis presented above.

The critical differences identified above are the relatively low nutrient levels, c. 100 μg SRP L^-1^, at which nutrients are no longer limiting to benthic algal growth and, hence, above this threshold there is always the potential for epiphytic algae to out-compete submerged macrophytes, if they can coat the plants sufficiently thoroughly for long enough to reduce light below tolerable conditions.

Where other factors also negatively influence light availability, either through brownification of water, suspended sediment load, or shading from riparian, emergent, or floating-leaved species, then eutrophication impacts and the loss of sensitive submerged species are more likely to be manifested.

We postulate less competitive submerged macrophyte species can also be excluded by competitive submerged macrophyte species at relatively low nutrient levels. The evidence for this is based on the dominance of many river systems by competitive species down to relatively low nutrient levels but a focused analytical study is required to confirm this assertion. Competitive emergent species along the borders of rivers increase in abundance (and grow out into the stream profile) at the expense of small amphibious species (e.g., *Veronica, Myosotis* species, etc.) that grow in the ecotone between land and water in natural streams.

In rivers of moderate slope, competitive submerged macrophytes may persist and thrive at high nutrient levels as hydraulic conditions are rarely suitable for the comprehensive coating of epiphytic algae necessary to outcompete the submerged aquatic plants. While there is field evidence for the persistence of submerged species at higher nutrient levels in moderate sloped systems, the interaction with algae remains to be tested. Previous findings suggest that species with apical growth points increase in abundance with increasing eutrophication probably because they concentrate biomass in upper waters where light availability is best (Baattrup-Pedersen et al., 2015). So along a gradient in nutrients, compositional patterns change in favor of species with apical growth (and/or low light compensation points).

We postulate that the habitat template set by instream hydraulic conditions form the basis for differential responses of macrophyte communities to nutrient enrichment. The evidence for the importance of instream hydraulic conditions and determining the suitability of a river reach for macrophytes is well supported. The differential response of macrophyte communities has yet to be confirmed empirically although it is consistent with analysis of large national datasets of macrophyte distribution from the United Kingdom. Specifically, we suggest that in steep sloped systems there will be little change in the bryophyte community following eutrophication, as its growth is limited by its naturally slow growth rate and the availability of suitable colonization sites. Competition between bryophyte species is, therefore, likely to be limited. When high nutrient concentrations do occur algae may develop in discrete areas where they can tolerate the shear stresses but will not persist past periods of stable flow, being washed out episodically. There is very limited potential for other submerged macrophytes or floating or emergent species to dominate as hydraulic conditions are unsuitable.

In channels of moderate slope, where, depending on the geology of the area, either gravel or sandy substrates can dominate, rheophilic submerged species dominate instream under natural conditions but less competitive species co-occur. Where hydraulic conditions are suitable, smaller stands of emergent and occasionally floating-leaved species occur but their distributions are curtailed by the physical suitability of the habitat. Eutrophication manifests itself as the competitive exclusion of the less competitive submerged species and an increase in density and cover of competitive submerged species such as the rheophilic Batrachian *Ranunculus* species. It should be noted that such moderate slope channels can be subject to flood flows that can reset communities and open areas for colonization by opportunistic species, such as the *Callitriche* species. One can postulate that this mechanism could significantly alter the presentation of a “typical” eutrophic system by providing space and opportunity for a range of macrophyte species. Intermediate sized streams are naturally disturbed systems and diversity is likely to be maintained by regular disturbances (thereby acting against the predominance of a few eutrophic species).

In channels on gentle slopes, we suggest eutrophication can progress in a manner analogous to lake eutrophication where the hydraulic conditions are suitable for the competitive exclusion of all submerged species through competition with epiphytic algae and/or phytoplankton. As lowland reaches of rivers, which are typically on gentle gradients, are also subject to suspended sediment loads, the light climate can be particularly challenging. There is also the possibility under these conditions that floating-leaved and emergent vegetation can out-compete submerged species by shading them, to the limits of their physical habitat. Water depth will limit such interactions, as both emergent and floating-leaved species will only persist to a limited depth, typically <3 m. It should be noted, however, that in a large Europe-wide study, there were no indications of an increase in floating-leaved species in response to eutrophication found, only apical growth (Baattrup-Pedersen et al., 2015); hence, the hypothesis needs empirical confirmation before it should be accepted.

The three scenarios, described above, should be considered as points along a continuum of hydraulic conditions, macrophyte assemblage structure and changing responses to eutrophication.

Travel time and its influence on the dominance of benthic or pelagic primary production is a confirmed key consideration and require further confirmation from new data collection. However, the importance of channel form and areas of slow flow in providing areas for phytoplankton numbers to increase must be considered in future studies.

## Concluding Remarks and Future Perspectives

### Implications for Management

#### Setting Ecological Targets and Associated Nutrient Reduction Levels

Setting ecological targets for eutrophication remediation has been hampered by a lack of time-series data illustrating changes in communities over time as they have become eutrophic and then recovered to pre-eutrophication conditions. While lake ecologists have been able to use fossils preserved in sediment to reconstruct the ecology of lake environments, for fluvial environments, the same process cannot be undertaken. For rivers, ecologists are reliant on studies by early naturalists to provide early data; some of the strongest evidence comes from Denmark (Sand-Jensen et al., 2000; Baattrup-Pedersen et al., 2008). Data were available to allow a comparison of the macrophytes communities of 13 streams sampled in the modern day, 1997 and pre-intensive eutrophication in 1896. It suggests that the *Potamogeton* vegetation, more commonly known as pondweed, has become less diverse, and communities are now dominated by species resistant to frequent disturbance through a high dispersal capacity. Disturbance is related to both physical modifications, including weed cutting, and eutrophication. These pondweed species are primarily submerged species which, for the most part, inhabit very similar hydraulic conditions where stream power and shear stresses are moderate or low.

Ecologists and river managers have, therefore, been required to set standards for recovery based on space for time substitution data, i.e., where a river macrophyte community not stressed by nutrient enrichment is identified as a target community for a site that is subject to nutrient enrichment but is in other respects a close match to the reference site. Sophisticated statistical approaches, adopted from river invertebrate assessment techniques, such as RIVPACS, can be applied to make use of data from multiple sites to set targets (Wright et al., 1998).

While these approaches have proved helpful, additional issues have arisen. The approach lacks the certainty of a known intervention having a desired outcome in terms of ecological improvement. For example, tertiary treatment has been included in many sewage treatment works with significant reductions in phosphorus concentrations in stream. However, the desired return of macrophyte vegetation has not been observed. The quantitative parameterization and confirmation of the responses of macrophytes to eutrophication, as described in the revised conceptual model, will help set realistic recovery targets to be set and help develop indicators of recovery trajectories. This work will inevitably require focused studies.

In Europe the EU Water Framework Directive (WFD) requires member states to judge water quality on the ability of their freshwater systems to support good ecological quality, including appropriate and diverse macrophyte communities. This has stimulated a substantial restoration effort including the reduction of diffuse and point source nutrient pollution. There is concern that the methods used in WFD monitoring to record aquatic macrophytes are not sensitive to eutrophication and that by inference, this could be conflated with the idea that macrophytes are not themselves sensitive or damaged by nutrient enrichment. An open discussion is now required to re-appraise these methods in light of our improved understanding of the damage done to macrophyte communities by eutrophication and the nutrient thresholds associated with key improvements in macrophyte assemblages.

Hilton et al. (2006) suggested that measuring the biomass of epiphytic algae would be a useful indicator of eutrophication. In lakes, monthly, and ideally more frequent, monitoring of primary production, is recommended. Given the nature of the fluvial environment and its control on all benthic algal production through physical removal, an even higher frequency of monitoring would seem appropriate, with daily measurements of algal biomass a sensible target. Such measures would require that new monitoring equipment and patents are in place, for such equipment and prototypes are under development. Such data would allow systems most suitable to rehabilitation to be targeted, i.e., where shear stresses already help to mediate algal dominance. Such targeted restoration requires an appreciation of the role eutrophication plays in multiple-stressed systems and the application of numerical modeling to facilitate management.

#### Addressing Eutrophication in Systems Subject to Multiple Stress

An emerging area of research is that on river systems subject to multiple stress. These studies focus on multivariate analyses of large monitoring datasets. The most comprehensive data on the extent of multiple stress comes from Europe where about half of all water bodies are subject to nutrient enrichment. Other stresses on systems include hydromorphological alterations, damming, channelization, and routine channel maintenance. There is strong spatial structuring, with lowland rivers most commonly subject to nutrient pollution and subject to channelization and are isolated from their floodplains (Schinegger et al., 2012). This pattern is likely to be found in other continents where intensive land use and urbanization are both associated with drainage, flood regulation, and excess nutrient delivery to water courses. Trait-based analytical approaches have proven successful in determining the effect eutrophication has on macrophyte communities in isolation and in the context of hydromorphological pressures caused by routine channel maintenance (Baattrup-Pedersen et al., 2015, 2016). Specific traits could distinguish hydromorphological degradation (free-floating, surface; anchored floating-leaves; anchored heterophylly) from eutrophication (free-floating, submerged; leaf area).

The importance of travel time and the influence of slow water zones in providing inoculum of phytoplankton to the main river flow is critical in the context of river restoration. The physical restoration of rivers is becoming increasingly popular (Friberg et al., 2016, 2017), but little consideration has been given to the impact on systems that are already subject to eutrophic stress. Equally, as most physical restoration projects involve small stretches of river, little thought has been given to the potential for cumulative impact. As many of the approaches involve the factors that will effect travel time, such as weir removal and the re-meandering of channels, it is suggested that due consideration is given to the potential for exacerbating the possibility of inducing phytoplankton production.

#### Potential for Numerical Modeling

When developed and tested with an appropriate detail of field- and laboratory-based measurement, empirical and process-based models of macrophyte presence, abundance, biomass, and succession are useful to help test hypotheses regarding the relationships between plant community development and the controlling abiotic and biotic factors, and to explore how aquatic plant communities will likely respond to environmental change and support catchment management (Gurnell, 2014; Wood et al., 2015). Whilst conceptual models of macrophyte growth and succession exist (Mainstone and Parr, 2002; Hilton et al., 2006; Hossain et al., 2017), computational models are relatively rare compared to those for river flow and water quality with most instream biological models focused on phytoplankton bloom development (Kowe et al., 1998; Chapra et al., 2007; Whitehead et al., 2015). Models of other components of a river ecosystem, such as the interaction between the benthic and suspended algae and/or macrophyte-epiphyte interactions exist, but progress has been limited by insufficient data to build both spatially explicit models that focus on community composition or dynamic models that simulate how macrophyte biomass and succession change over time (Bartell et al., 1999; Schol et al., 1999; Wade et al., 2001, 2002a,b; Greenwood and Rosemond, 2005; Robson and Webster, 2006; Park et al., 2008; Sourisseau et al., 2008; Lazar et al., 2016). This lack of data is particularly acute when attempting to separate the effect of individual stressors on primary production and community succession, when trying to move from an understanding of how an individual plant might respond to an overall population or plant community response, and when exploring trophic interactions. The original hypotheses of Hilton et al. (2006) have been tested using a computational model but in a theoretical way due to issues around finding sites with sufficient data to quantify the response of all the primary producers (Lazar et al., 2010). Lazar et al. (2016) provide a computational model within which to test field and laboratory data, and highlight the data and key relationships that still need to be robustly established. Significant advances in predictability can be made if the core concepts identified in this model are parameterized.

Progress has been made with plant community modeling in wetlands at a national scale (Acreman et al., 2009) and with the simulation of lake cyanobacteria and phytoplankton community development using Bayesian Belief Networks and process-based models, respectively (Elliott et al., 2010; Moe et al., 2016), all based on model development and testing using intensive measurement. In addition, there is a general understanding of the “top-down” controls on phytoplankton and macrophyte growth in rivers with the hypotheses of Hilton et al. (2006), now supplemented with information on potential thresholds in light, temperature, flow, pH, and nutrient concentrations that prevent or initiate bloom development (Bowes et al., 2016). The subtle dynamics of grazing (Keckeis et al., 2003) and microbial pathogenesis (Mayali and Azam, 2004; Maier and Peterson, 2017) are only beginning to emerge. Given this understanding of the key controlling factors to focus on, an opportunity to bring progress to the development of river ecosystem models that include macrophytes and their relationship with phytoplankton can be made through the measurement of growth and succession along stressor gradients, including channel geomorphology (Tena et al., 2017). Studies that investigate the cause–effect relationships of aquatic functional groups will support more robust empirical and process-based models (Hering et al., 2006).

If these stressor–response relationships can be specified from field observations supplemented by laboratory studies, then there is opportunity to embed them into existing catchment, national, regional, and global scale dynamic models (Lindstrom et al., 2010; Seitzinger et al., 2010). Hydrological and water quality models are being developed and applied at this range of scales and the data that describe the climate and weather, land cover and use, soil moisture status and even geomorphology are now becoming available from remote and *in situ* sensing. There is probably no need to run such models in real-time to investigate macrophyte dynamics, but these models can be run off-line to simulate how extreme weather, land cover and use, and geomorphological change may affect macrophyte growth and succession. However, since macrophytes have an important impact on flood-wave propagation (De Doncker et al., 2008), if macrophyte models are coupled with large-scale hydrological models, flood-prone zones could be identified. New field observations across a range of sites that cover stressor gradients would not only allow the structural and parameter refinement of numerical models, but would also enable the use of space for time substitution to see how communities may evolve under the projected stressor change. Finally, there is also opportunity to compare the response of freshwater plants with terrestrial, riparian, and coastal plant communities over the long term to identify guild strategies and thresholds for community change and recovery as stressors change (Haase et al., 2018).

## Concluding Remarks

Our knowledge and understanding of the responses of aquatic plant to eutrophication in rivers has accelerated in the last decade. A picture is emerging of a more refined conceptual model that is suitable for empirical testing and has the potential to be developed into numerical models.

These developments bring clarity to management approaches and indicate the future direction of aquatic plant conservation. In the following decade, we can expect more focus in the selection of rivers for rehabilitation from eutrophication. Numerical modeling will allow local, national and global strategies to combat eutrophication impacts to be developed. There is a clear sense that the supporting science, while still under development, is now well placed to improve aquatic plant management globally.

## Author Contributions

MO instigated the production of the paper with MB and wrote 60% of the text with IG helping with a review of the relevant scientific literature as well as the proofreading of the paper. MB contributed 15% to the general development of concepts and wrote the section on nutrient and light limitation. AW led the section on modeling with contributions from AF and AL, 15% in total. RS provided 10% of the supporting text. IB created the diagrams. AB-P helped develop a coherent and consistent narrative in the paper.

## Conflict of Interest Statement

The authors declare that the research was conducted in the absence of any commercial or financial relationships that could be construed as a potential conflict of interest.
